# Knowledge and practices of supervisors on the performance management and development system at rural primary health care facilities in the Limpopo Province

**DOI:** 10.4102/phcfm.v8i1.1236

**Published:** 2016-12-02

**Authors:** Rosemary H. Mashego, Linda Skaal

**Affiliations:** 1 Department of Public Health, University of Limpopo, South Africa

## Abstract

**Background:**

The South African government has introduced Performance Management and Development System (PMDS) as a tool to monitor and manage the performances of health institutions, in order to improve service delivery within primary health care settings. The aim of the study was to determine the knowledge and practices of supervisors regarding PMDS in primary health institutions of the Limpopo Province.

**Materials and Methods:**

A cross-sectional, descriptive, quantitative study was used. A total of 117 participants were sampled using stratified random sampling technique and a questionnaire was used to collect data. Statistical Package for Social Sciences (SPSS) version 22.0 was used to analyse both descriptive and inferential statistics.

**Results:**

Generally all the respondents had an average (65.8%) understanding of the PMDS processes including the purpose and their roles as supervisors. However, a gap exists between the theoretical knowledge and the actual ability to practise PMDS which was found to be at 52%. There are areas of weakness that still need attention, such as unavailability of PMDS guidelines and lack of training of both supervisors and employees on PMDS.

**Conclusion:**

This study highlights the problem of lack of knowledge and skills, unavailability of PMDS policy and poor induction into PMDS. To improve the knowledge and ability to supervise PMDS, proper induction of all PMDS supervisors and periodic in-service training should be done; reference materials, the PMDS policy manuals, are to be made available in the facility and all supervisors be orientated on how to use these manuals.

## Background

Performance Management and Development System (PMDS) is a strategy used for tracking and evaluating the performance of employees in an organisation.^1^

PMDS is a widely used system for managing performance globally. There has been a shift from performance appraisal (PA) which was mostly used by private companies in the United States, Norway and many more countries for decisions on merit awards, promotions and salary increases.^2^ Several factors such as global competition for excellence, increased customer expectation for quality products, and so on, have influenced the global markets to re-think a more comprehensive and holistic strategy to manage performance.^3^ According to Bernthal et al.,^4^ success in the use of PMDS was noticed in a wide range of international private companies in the United States, Europe and Australia. Such success include improved employee productivity, increased sales, quality of products, services as well as improvement of skills, since PMDS is linked with monitoring of service and skills development.^4^

Similarly, in South Africa, there were factors that motivated the government to improve the public services through a proper system of performance management. According to Kanyane,^5^ since 1994, the South African public became aware of their rights, and this has increased their expectations and demand for quality services. In order to meet these demands, the government needed a public service that will perform effectively to create and sustain a better life for its citizens. Some of the policies that supported transformation, among others, were the Public Service Regulation, Chapter 1, Part Viii^6^; the White Paper on Transformation of Public Service, 1995^7^; and the Skills Development Act, 1998,^8^ which provides for training and development of workers.

PMDS is designed such that it allows for continuous assessment and timeous identification of performance gaps, and institution of corrective measures. Possession of knowledge and skills are a requirement to build an effective performance management system. There are PMDS benefits for both the employee and the employer. Employees are rewarded for outstanding performance through pay progression, cash bonuses and skills development. The employer on the contrary is able to fulfil the service delivery mandate through improved performance by employees.^1^

Although PMDS is believed to be a good tool to evaluate employee performance, major challenges exist in its implementation. Since its inception in 2002 in the Limpopo Department of Health, PMDS supervisors in the Greater Tzaneen sub-district still express frustrations during the signing of performance contracts and performance reviews. This challenge is nationwide, as was revealed in the studies conducted in various provinces in South Africa, including the Limpopo Province.^9,10^ So far, it is not known why these challenges persist. This study focused on investigating the knowledge and practices of PMDS among supervisors in primary health care institutions, in the Greater Tzaneen sub-district, which is a rural area in the Limpopo Province.

## Methods

A quantitative, cross-sectional design was used. Ethical clearance was obtained from the Medunsa Research and Ethical Committee (MREC) of the University of Limpopo, Provincial Department of Health Research Committee.

### Study participants

A total of 117 nurses, comprising assistant managers (6), operational managers (20) and professional nurses (91) were sampled from a population of 274 nurses from the primary health care facilities in Greater Tzaneen. The sample size was calculated using the following formula:

$$S=\frac{X^{2}NP\left(1-P\right)}{d^{2}\left(N-1\right)+X^{2}P\left(1-P\right)}$$ [Eqn 1]

@ *1.96 for 95% confidence level, where population proportion is assumed to be 0.5 (50%)and degree of accuracy (5%) is expressed as a proportion (0.05).*

All these participants were of supervisory cadre and had 1 to > 4 years of service as supervisors. The respondents were recruited via telephone and verbally, and those who agreed were sampled using stratified random sampling technique to reach the desired sample size per stratum, according to professional rank, health centres and sub-district offices. Ethical clearance was obtained from a university research ethics committee and permission from Limpopo Provincial Health Research Committee.

### Data collection

Data were collected using a structured questionnaire adopted from the existing questionnaires for PMDS.^9,11^ The questionnaires comprised three parts: demographic information, knowledge of PMDS and practice or implementation of PMDS. Reliability of the instrument was ensured through pilot testing. Validity was ensured by using previously validated instruments from the research studies and supervisor and peers for content validity.

### Data analysis

Data were coded and entered into the Statistical Package for Social Sciences (SPSS) version 22.0 for analysis. A total of 16 questions were used to establish the level of knowledge: the scales for knowledge were as follows: purpose of evaluation, processes used in evaluation and supervisor’s responsibilities. Furthermore, overall knowledge was classified into poor (< 50%), fair (50% – 60%), good (61% – 74%); and excellent (≥ 75%). For descriptive analysis, frequency distributions, central tendencies and mean and standard deviations were calculated. To determine the association between socio-demographic factors, knowledge and practice of PMDS, Chi-square analysis was used where a *p*-value of < 0.05 was considered statistically significant.

## Results

Table 1 shows that the majority of the respondents had a basic nursing diploma (66.7%). Also, 41% of respondents had worked for > 10 years and 59% had worked for ≤ 10 years. Most respondents reported that they had supervised PMDS for 4 years or more (41.9%) while 58.1% had supervised for ≤ 4yrs.

**TABLE 1:** Socio-demographic profile of the participants (% in columns).

Variables *N* = 117	Frequency	%
**Gender**		
Male	9	7.70
Female	108	92.30
**Current position**		
Professional nurse	91	77.8
Operational manager	20	17.1
Assistant manager	6	5.1
**Years of experience**		
≤ 10 years	69	59.0
> 10 years	48	41.0
**Number of years supervising PMDS**		
≤ 4 years	68	58.1
> 4 years	49	41.9

Table 2 shows that the majority of the respondents reported the availability of job descriptions (73.5%) and operational plans (75.2%) in their facilities. Also, about 41.9% reported that there were no PMDS manuals in their facilities. The majority of the respondents (70.9%) reported that they have never attended workshops on PMDS, with only 27.4% reporting in the affirmative. The respondents also reported that 46.2% of the employees had received orientation on PMDS, while 32.5% were not orientated and 21.4% were not sure. Also, 59% of the respondents reported that they had provided their employees with the opportunity to attend skills development trainings.

**TABLE 2:** Availability of PMDS documents and staff training (% in columns).

Variables *N* = 117	Yes *N* (%)	No *N* (%)	Not sure *N* (%)
Job descriptions available	86 (73.5)	23 (19.7 )	8 (6.8)
Operational plan available	88 (75.2)	18 (15.4)	11 (9.4)
PMDS policy manual available	37 (31.6)	49 (41.9)	31 (26.5)
PMDS supervisor attended workshop on PMDS	32 (27.4)	83 (70.9)	2 (1.7)
The staff have been trained on PMDS	54 (46.2)	38 (32.5)	25 (21.4)
Employees undergo training programme to close skills gap	69 (59.0)	30 (25.6)	18 (15.4)

**FIGURE 1:**
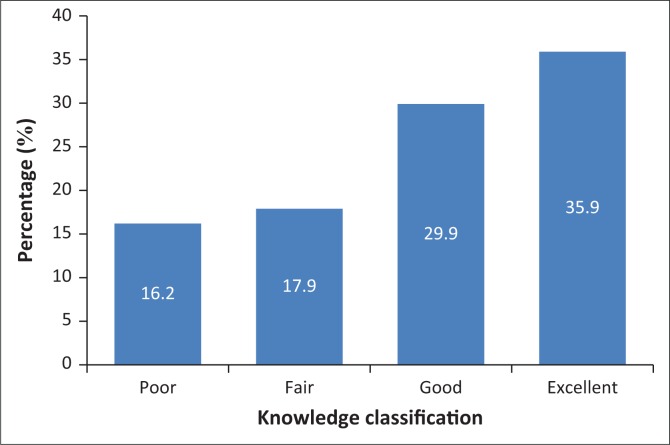
Knowledge of respondents about PMDS processes.

Figure 1 shows the respondents’ knowledge of PMDS processes, these results on knowledge were based on respondents self-report on their knowledge of PMDS processes. About two-thirds (65.8%) had good –excellent knowledge and 34.2% fair–poor knowledge.

**FIGURE 2:**
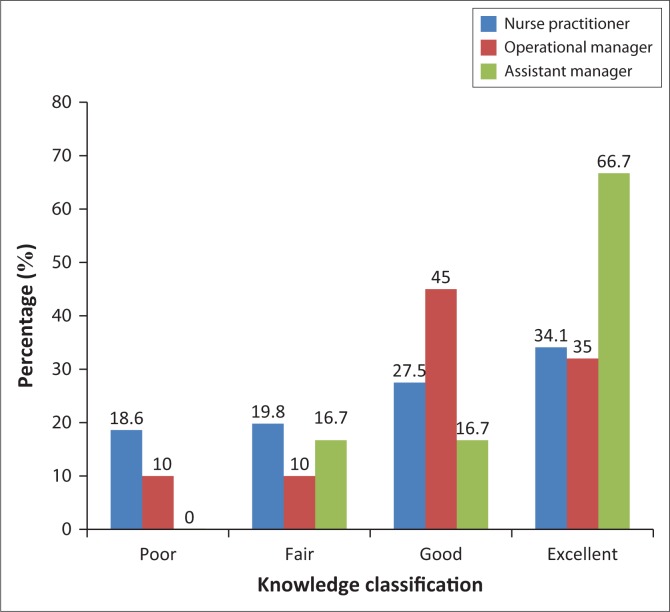
Knowledge of PMDS processes according to the respondent’s professional position.

Figure 2 shows that the majority of assistant managers had excellent knowledge (66.7%) compared with operational managers (35%) and professional nurses (34.1%). Also, 18.6% of professional nurses and 10% of operational managers were found to have poor knowledge, while none of the assistant managers had poor knowledge on PMDS. However, there is no significant association between knowledge of PMDS and the respondent’s position, *p* = 0.071.

**FIGURE 3:**
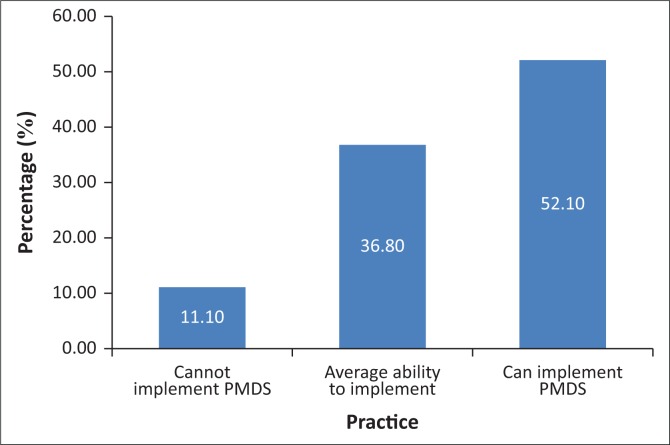
PMDS implementation practices scores.

Figure 3 shows that most of the respondents (52.1%) were able to implement PMDS; 36.8% had average ability, while only 11.5% were lacking the abilities to implement PMDS.

Table 3 shows that there is no significant association among all the categories (*p* > 0.05). Also, there was no significant association between the years of experience and ability to implement PMDS (*p* > 0.05) and no difference between the respondents who attended training and those who did not in relation to their ability to implement PMDS.

**TABLE 3:** Ability to implement PMDS by professional category, years of experience and training.

Variables *N* = 117	Cannot implement *N* (%)	Average ability to implement *N* (%)	Can implement *N* (%)	*p*
**Professional categories**				
Professional nurse	11(12.2)	33(36.6)	46(51.2)	*X*^2^ = 3.130
Operational manager	2(10.0)	6(30.0)	12(60.0)	*p* = 0.830
Assistant manager	0	4(66.7)	2(33.3)	
**Years of work experience**				
< 5 years	2(7.5)	11(40.7)	14(51.8)	*X*^2^ = 6.614
6–10 years	2(4.7)	19(45.2)	21(50.0)	*p* = 0.531
> 10 years	9(18.7)	13(27.0)	26(54.1)	
**Training on PMDS implementation**				
No training	2 (6.2)	11 (34.4)	19 (59.3)	*X*^2^ = 1.447
Attended training	11 (12.9)	32 (37.6)	42 (49.4)	*p* = 0.241

PMDS, performance management and development system.

## Discussion

Professional nurses constituted the majority of participants in this study while close to a quarter were managers. Females were the most dominant group among the participants, according to the South African Nursing Council (SANC) statistics; females constituted 94%of professional nurses while males were only 6% (SANC 2007).

Although these nurses differ in qualification, ranks and years of experience, in terms of PMDS supervision, they were all eligible to supervise anybody lower in rank, as stipulated in the PMDS circular no. 67 of 2010, 6.2.10. Because of the small number of assistant and operational managers, the situation warrants that the supervision of most of the PMDS is assigned to professional nurses as shown in this study.

Our results revealed that most staff had less than 10 years of work experience. According to Letsoalo,^12^ the supervisors’ years of experience influences how they perform their duties; correspondingly, their skills in assessing staff performance using PMDS also increases. Paile^10^ also suggested that the professional position of the supervisor, years of professional experience and the number of years of exposure to PMDS supervision may determine the success of PMDS implementation. Our results showed that the majority of managers and assistant managers had good–excellent knowledge compared with professional nurses and there was a significant association between professional category and knowledge.

An effective PMDS is one that is aligned with the vision and mission, employee job description as well as the strategic and operational plans of the department. These documents describe what the department needs, and how, to achieve its objectives. In order for these documents to be of good use, they must be communicated to both the PMDS supervisors and the subordinates to ensure that they are used effectively. PMDS policy manual serves as a standard operating procedure, which guides in PMDS implementation, from the signing of the contract through to performance evaluation.^1^ Our study found that two-thirds of the respondents did not have the policy manual in their facilities, they depended on colleagues from other facilities as a source of reference and some did not refer at all. The absence of the PMDS policy manual poses a challenge in measuring the quality of the system; we therefore deduced that PMDS implementation in these facilities was by trial and error for some supervisors who never received training on the implementation of PMDS and for those who did not poses the PMDS manual.

Training on PMDS of both the supervisor and the employee is crucial. According to Pulakos^13^ and Smith,^14^ skills acquisition has a positive effect on the ability to implement PMDS; the success of PMDS implementation depends on the skills of the supervisor. A study conducted by Malefane^15^ in BaPhalaborwa, the Limpopo Province, confirms the positive linkage between training and good performance. It is therefore imperative that both the supervisor and the employee undergo training to assist them to understand PMDS processes.

This is supported by findings from a study conducted by Maluleke^9^ which revealed that the majority of the supervisors believed that training of employees improves their performance, which, in turn, ensures that employees score well on PMDS. On the contrary, our study found that the majority of the PMDS supervisors reported that they never received any training on PMDS, especially professional nurses, while managers reported that they did receive training. However, our study found no significant statistical difference in the ability to implement PMDS between the respondents who reported that they had attended training and those who did not attend training.

The impression of these results is that most of the supervisors were operating on hearsay, while they are expected to be experts in order to be able to coach and mentor others. Training was not considered a priority in PMDS, especially to the professional nurses, most of whom have less years of work experience. The vision and strategic development of any company lies in the ability of supervisors to carry out the managerial functions which include ability to influence, inspire and develop subordinates that serve under him/her. Inadequate knowledge is a hindrance to effective implementation of one’s duties as a manager or supervisor.^16^

The good practice of PMDS is determined by the extent to which it complies with the standards of operation stipulated in the PMDS manual. The results revealed that only one half of the respondents were able to competently implement PMDS. This implies that the other half were operating sub-optimally or poorly in terms of using PMDS as a tool to evaluate service delivery by subordinates, which can lead to employees being over-scored or under-scored in terms of performance of their duties. Supervisors are responsible for ensuring quality service delivery. So, if they themselves do not understand the very quality assurance tool they are supposed to use to measure service delivery, they will never know whether their subordinates are performing sub-optimally or not. It is therefore recommended that the Limpopo Provincial department should ensure that all supervisors receive training on how to use PMDS, in order for them to correctly implement it to monitor service delivery.

## Conclusion

This study highlights the problem of lack of knowledge and skills, lack of PMDS policy and poor induction into PMDS. Most of the supervisors are professional nurses who were deprived of proper induction and training although they form a pool which supplies PMDS supervisors to the department of health. Our findings highlighted the need for training of supervisors to improve their knowledge, skills and implementation.

### Limitations of the study

The targeted population covered only one sub-district; therefore, it is difficult to generalise the findings to all PMDS supervisors in the Limpopo Province. However, previous studies have revealed poor knowledge in PMDS in other areas of the Limpopo Province.

### Recommendations

We recommend that proper induction be provided to all potential PMDS supervisors and they be equipped with the requisite skills to supervise their subordinates.

There should be regular workshops and in-service training to all PMDS supervisors.

Lastly, we recommend that there must be implementation of quality control measures to all performance instruments and completed review forms to reinforce compliance to the PMDS policy. All managers should ensure that PMDS policy manuals are made available for all employees.

### Strengths

The most important strength of this study is the fact that it highlights the need for supervisors to be trained in order to strengthen health systems. This will in turn improve service delivery which in turn improves the monitoring of staff within the primary healthcare system.
